# Triplex Crystal Digital PCR for the Detection and Differentiation of the Wild-Type Strain and the MGF505-2R and I177L Gene-Deleted Strain of African Swine Fever Virus

**DOI:** 10.3390/pathogens12091092

**Published:** 2023-08-28

**Authors:** Kaichuang Shi, Kang Zhao, Haina Wei, Qingan Zhou, Yuwen Shi, Shenglan Mo, Feng Long, Liping Hu, Shuping Feng, Meilan Mo

**Affiliations:** 1 College of Animal Science and Technology, Guangxi University, Nanning 530005, China; zhaokang0519@163.com (K.Z.); shiyuwen2@126.com (Y.S.); 2 Guangxi Center for Animal Disease Control and Prevention, Nanning 530001, China; weihaina@sina.cn (H.W.); zhouqingan1@163.com (Q.Z.); moshl_2015@126.com (S.M.); longfeng1136@163.com (F.L.); hu.liping@foxmail.com (L.H.); fsp166@163.com (S.F.)

**Keywords:** African swine fever virus (ASFV), multiplex crystal digital PCR (multiplex cdPCR), multiplex real-time quantitative PCR (multiplex qPCR), gene-deleted strain, wild-type strain

## Abstract

African swine fever (ASF) is a severe and highly contagious viral disease that affects domestic pigs and wild boars, characterized by a high fever and internal bleeding. The disease is caused by African swine fever virus (ASFV), which is prevalent worldwide and has led to significant economic losses in the global pig industry. In this study, three pairs of specific primers and TaqMan probes were designed for the ASFV B646L, MGF505-2R and I177L genes. After optimizing the reaction conditions of the annealing temperature, primer concentration and probe concentration, triplex crystal digital PCR (cdPCR) and triplex real-time quantitative PCR (qPCR) were developed for the detection and differentiation of the wild-type ASFV strain and the MGF505-2R and/or I177L gene-deleted ASFV strains. The results indicate that both triplex cdPCR and triplex qPCR were highly specific, sensitive and repeatable. The assays could detect only the B646L, MGF505-2R and I177L genes, without cross-reaction with other swine viruses (i.e., PRRSV, CSFV, PCV2, PCV3, PEDV, PDCoV and PRV). The limit of detection (LOD) of triplex cdPCR was 12 copies/reaction, and the LOD of triplex qPCR was 500 copies/reaction. The intra-assay and inter-assay coefficients of variation (CVs) for repeatability and reproducibility were less than 2.7% for triplex cdPCR and less than 1.8% for triplex qPCR. A total of 1510 clinical tissue samples were tested with both methods, and the positivity rates of ASFV were 14.17% (214/1510) with triplex cdPCR and 12.98% (196/1510) with triplex qPCR, with a coincidence rate of 98.81% between the two methods. The positivity rate for the MGF505-2R gene-deleted ASFV strains was 0.33% (5/1510), and no I177L gene-deleted ASFV strain was found. The results indicate that triplex cdPCR and triplex qPCR developed in this study can provide rapid, sensitive and accurate methods for the detection and differentiation of the ASFV B646L, MGF505-2R and I177L genes.

## 1. Introduction

African swine fever (ASF) is an acute, highly contagious infectious disease that causes hemorrhagic fevers in infected pigs, and the mortality rate might reach almost 100% [1]. The etiological agent, African swine fever virus (ASFV), is an enveloped, double-stranded DNA virus that belongs to the family *Asfarviridae*, and it is the only member of the genus *Asfivirus* [2]. The ASFV viral particle size ranges from 260 to 300 nm in diameter and contains a 170–194 kb genome encoding over 160 open reading frames (ORFs) with conserved central regions and variable ends [3,4]. ASF was first discovered in Kenya in 1921, and it subsequently spread to Europe, where it was first detected in Portugal in 1957. Subsequently, ASF was found in the Caucasus and southern Russia in 2007 [5,6]. Currently, outbreaks of ASF are ongoing in various regions of the world, including Africa, the Caucasus, Eastern Europe, the Russian Federation, Asia and Latin America [7,8]. ASF was first reported in China in August 2018 [9] and quickly spread to most provinces within a very short time [10,11]. Thereafter, other Asian countries, including Mongolia, South Korea, Vietnam, Laos, Cambodia, the Philippines and Indonesia, have also reported ASF [12,13]. ASF has caused significant economic losses in the global pig industry.

To date, 24 genotypes of ASFV based on the B646L gene have been discovered around the world, and most genotypes have spread within the African continent. Only genotype I and genotype II strains of ASFV have been reported outside Africa. The genotype I strain of ASFV was first identified in Portugal in 1957, and the genotype II strain of ASFV was first discovered in Georgia in 2007 [5,14,15]. Today, genotype II ASFV is the dominant genotype that is prevalent in various countries around the world [15,16]. Since the first report of ASF in China in 2018, most prevalent strains of ASFV have been found to be genotype II [17,18], but genotype I strains of ASFV have been found in several provinces (Anhui, Shandong and Guangxi provinces) of China [17,19,20]. As the predominant strains of ASFV, the genotype II ASFV strains have posed a serious threat to the healthy development of the Chinese pig industry. In addition, several provinces in China have identified naturally variant ASFV strains that lack the MGF360-505R and EP402R genes [21]. These strains demonstrated reduced virulence, and the infected pigs exhibited low morbidity and mortality rates. Genotype I strains that lack the MGF360 and MGF505 gene families have been discovered in Guangxi province, southern China [17]. Currently, the emergence of gene-deleted strains of ASFV has introduced new challenges to the prevention and control of ASF [21,22]. In addition, many scientists have reported some ASFV vaccine candidates that lack some of the virulence genes. By deleting one or several genes of MGF505-1R, MGF505-2R, MGF505-3R, MGF360-12L, MGF360-13L, MGF360-14L, CD2v, 9GL, DP148R and UK, scientists generated six novel gene-deleted vaccine strains, and these strains have demonstrated good safety and efficacy [23]. Several studies have confirmed that strains lacking the MGF360-505R and EP402R genes can be used as ideal candidates for developing live attenuated vaccines [24,25,26]. It is noteworthy that an ASF vaccine candidate, which has been genetically modified to have a deletion in part of the I177L gene, has been demonstrated to provide protection to pigs against highly pathogenic strains of ASFV that are presently circulating in Europe and Asia [27,28,29,30]. The attenuated vaccine that utilized the I177L gene-deleted strain was officially approved for commercial use in Vietnam in June 2022 (http://link.gov.vn/vtUM759t, accessed on 9 March 2023). This vaccine is the first commercial vaccine approved for clinical use for preventing ASF in the world [31]. Especially, the MGF505-2R and/or I177L gene-deleted strains of ASFV have aroused great interest and become the focus of attention in the pig industry. Therefore, it is very necessary to develop a rapid, sensitive and accurate method to detect and differentiate the wild-type strain and the MGF505-2R and/or I177L gene-deleted strains of ASFV.

Real-time quantitative PCR (qPCR) is a convenient, efficient, accurate and high-throughput technology and has been widely used to test viral nucleic acids [32]. Digital PCR (dPCR) is a powerful technology that has been developed in recent years, particularly in the field of microbiological research. Compared to conventional PCR and qPCR, dPCR does not require a standard curve for absolute quantitative analysis and is more accurate for the detection of the low number of DNA or RNA molecules [32,33,34]. To date, dPCR has been developed as an accurate method for the detection of ASFV, including droplet digital PCR (ddPCR) [35,36], crystal digital PCR (cdPCR) [37] and other similar technologies [38]. qPCR has been developed for the detection of ASFV [39,40,41], and several multiplex qPCRs based on the E296R, B646L or E183L genes of ASFV have been reported for the detection of genotypes I and II of ASFV [42,43,44]. In addition, several assays have been developed for the differential detection of the wild-type strains of ASFV and for the MGF505-2R, MGF-360-14L, CD2v, EP402R and/or I177L gene-deleted strains of ASFV [35,45,46,47,48]. In this study, triplex cdPCR and triplex qPCR, which target the ASFV B646L gene, MGF505-2R gene and I177L gene, were developed to detect and differentiate the wild-type strain and the MGF505-2R and I177L gene-deleted strains of the ASFV. The developed assays were used to test 1510 clinical tissue samples collected from Guangxi province, southern China, to validate the application of these assays.

## 2. Materials and Methods

### 2.1. Viral Strains

The vaccine strains of classical swine fever virus (CSFV, C strain), porcine circovirus type 2 (PCV2, SX07 strain), porcine reproductive and respiratory syndrome virus (PRRSV, TJM-F92 strain), porcine epidemic diarrhea virus (PEDV, SCJY-1 strain) and pseudorabies virus (PRV, Bartha-K61 strain) were purchased from Huapai Bioengineering Group Co., Ltd. (Chengdu, China). The positive clinical tissue samples of ASFV, PCV3 and porcine deltacoronavirus (PDCoV) were provided by the Guangxi Center for Animal Disease Control and Prevention (CADC), China. These viruses and samples were stored at −80 °C until use.

### 2.2. Clinical Samples

From January 2022 to December 2022, a total of 1510 clinical tissue samples from 1510 dead pigs were collected from different pig farms, slaughterhouses, farmers’ markets and harmless disposal sites in Guangxi province, southern China. The tissue samples from each pig included livers, spleens, kidneys and lymph nodes and were pooled and homogenized before being tested with the developed triplex cdPCR and triplex qPCR. The samples were transported to our laboratory at ≤4 °C within 12 h (from the death of the pig to the arrival of the samples at the laboratory) and were stored at −80 °C until use.

### 2.3. Design of Primers and Probes

Three pairs of specific primers and TaqMan probes were designed using the Primer Express Software v3.0 (ABI, Los Angeles, CA, USA), which targeted the conserved regions of the B646L, MGF505-2R and I177L genes, respectively. If the ASFV strain is positive for these three genes, it is the wild-type strain; if the ASFV strain is negative for the MGF505-2R and/or I177L genes, it is the MGF505-2R and/or I177L gene-deleted ASFV strain. The primers and probes are shown in Table 1.

### 2.4. Extraction of Nucleic Acids

Phosphate-buffered saline (PBS, pH 7.2) was added to the vaccines and the clinical tissue homogenates (20%, W/V), and the solutions were vortexed for 5 min and centrifuged at 12,000× *g* at 4 °C for 10 min. The total nucleic acids were extracted from the homogenized tissues or vaccine solutions using the GeneRotex 96 Automatic Nucleic Acid Extractor (TIANLONG Scientific, Xi’an, China) with Viral DNA/RNA Isolation Kit 4.0 (TIANLONG Scientific, Xi’an, China) according to the manufacturer’s instructions. The obtained nucleic acids were stored at −80 °C until use.

### 2.5. Construction of the Standard Plasmids

The DNA of ASFV was used as a template to amplify the targeted fragments of the B646L gene, the MGF505-2R gene and the I177L gene via PCR using the specific primers, respectively. The purified amplicons were ligated into the pMD18-T vector (TaKaRa, Dalian, China) and were subsequently transformed into *E. coli* DH5α competent cells (TaKaRa, Dalian, China). The positive clones were cultured at 37 °C for 20–24 h, and the plasmid constructs were extracted using MiniBEST Plasmid Extraction Kit Ver.5.0 (TaKaRa, Dalian, China), confirmed by sequencing. The recombinant plasmids were named pASFV-B646L-1, pASFV-MGF505-2R-1 and pASFV-I177L-1, respectively, and were used as standard plasmids in this study. They were stored at −80 °C until use.

The standard plasmids were quantified using a NanoDrop spectrophotometer (Thermo Fisher, Waltham, MA, USA) to measure their ultraviolet absorbance at 260 nm and 280 nm. Their concentrations were determined using the following formula: plasmid copy number (copies/μL) = (plasmid concentration × 10^−9^ × 6.02 × 10^23^)/(660 Dalton/bases × DNA length).

The sequences located at two ends of the ASFV MGF505-2R and I177L genes were artificially synthesized, respectively, by TaKaRa Biomedical Technology Co. Ltd. (TaKaRa, Dalian, China) and were then inserted into the pMD18-T vector (TaKaRa, Dalian, China) to construct the plasmids without the MGF505-2R gene (named pASFV-ΔMGF505-2R) and the I177L gene (named pASFV-ΔI177L), respectively. They were used as the MGF505-2R and I177L gene-deleted controls. The sequences of pASFV-ΔMGF505-2R and pASFV-ΔI177L are shown in Supplementary Table S1.

### 2.6. Optimization of the Reaction Conditions

The optimal conditions for triplex qPCR, including the annealing temperature and the concentrations of primers and probes, were determined using the QuantStudio 6 qPCR detection system (ABI, Carlsbad, CA, USA). The parameters used for amplification were as follows: pre-denaturation at 95 °C for 2 min, followed by 40 cycles of denaturation at 95 °C for 5 s and annealing and extension at 59 °C for 30 s. At the end of each cycle, the fluorescent signals were determined. In order to determine the optimal reaction conditions for triplex qPCR, the basic components were as follows: 12.5 μL of Premix Ex Taq (Probe qPCR) (TaKaRa, Dalian, China); 2.5 μL of the mixture of three standard plasmids with a final reaction concentration of 10^8^ copies/μL for each plasmid; 3 pairs of primers and corresponding TaqMan probes with different final concentrations (from 100 nM to 500 nM); and nuclease-free distilled water to a total volume of 25 μL. The ultimate reaction conditions were optimized to achieve the highest ΔRn and the lowest threshold cycle (Ct).

The Naica^TM^ sapphire crystal system (Stilla Technologies^TM^, Villejuif, France) was used to perform triplex cdPCR. The entire process was carried out within the Sapphire chip (Stilla Technologies^TM^, Villejuif, France). After thermocycling, the chips were transferred to Naica^TM^ Prism3 (Stilla Technologies^TM^, Villejuif, France) to image the FAM, VIC and CY5 detection channels. The concentrations of the templates were then determined using the Crystal Miner software (Stilla Technologies^TM^, Villejuif, France). The parameters used for amplification were as follows: 95 °C for 2 min; 45 cycles of 95 °C for 5 s; and 59 °C for 30 s. A total volume of 25 μL was used to determine the optimal reaction conditions of triplex cdPCR using the following basic systems: 12.5 μL of PerfeCTa Multiplex qPCR ToughMix (Quanta Biosciences, Gaithersburg, MD, USA); 2.5 μL of Fluorescein Sodium Salt (1 μM) (Apexbio Biotechnology, Beijing, China); a mixture of three pairs of primers and three probes of different final concentrations of 0.1–0.9 μL and 2.5 μL of the mixture of three standard plasmids with concentrations of 10^4^ copies/μL for each plasmid; and nuclease-free distilled water to a final volume of 25 μL.

### 2.7. Analytical Specificity Analysis

The DNA of ASFV, PCV2, PCV3 and PRV was extracted from the vaccine solutions or homogenized positive samples. The RNA of PRRSV, CSFV, PEDV and PDCoV was extracted from the vaccine solutions or homogenized positive samples, and it was then reverse transcribed to cDNA using the PrimeScript Ⅱ 1st Strand cDNA Synthesis Kit (TaKaRa, Dalian, China).

The DNA or cDNA of ASFV, PCV2, PCV3, PRV, PRRSV, CSFV, PEDV and PDCoV, as well as the recombinant gene-deleted plasmids pASFV-ΔMGF505-2R and pASFV-ΔI177L, was used to evaluate the specificity of the developed assays. The mixture of three standard plasmids was used as the positive control, and nuclease-free distilled water was used as the negative control.

### 2.8. Analytical Sensitivity Analysis

The standard plasmids pASFV-B646L-1, pASFV-MGF505-2R-1 and pASFV-I177L-1 were mixed at a ratio of 1:1:1 and were 10-fold serially diluted. The sensitivity of triplex cdPCR was analyzed using the mixed plasmids at concentrations from 2.0 × 10^5^ copies/μL to 2.0 × 10^−1^ copies/μL (final reaction concentration). The sensitivity of triplex qPCR was evaluated using the mixed plasmid at concentrations from 2.0 × 10^6^ copies/μL to 2.0 × 10^0^ copies/μL (final reaction concentration).

### 2.9. Repeatability Analysis

The standard plasmids pASFV-B646L-1, pASFV-MGF505-2R-1 and pASFV-I177L-1 were mixed at a ratio of 1:1:1 and were 10-fold serially diluted. The concentrations of 2.0 × 10^4^, 2.0 × 10^3^ and 2.0 × 10^2^ copies/µL (final reaction concentration) of each plasmid were used as templates to perform in triplicate for the intra-assay test and on three different days for the inter-assay test. The coefficients of variation (CVs) were obtained.

### 2.10. Detection of the Clinical Samples

A total of 1510 clinical tissue samples collected in Guangxi province in China from January 2022 to December 2022 were tested with the developed triplex cdPCR and triplex qPCR, and the coincidence rate and Kappa value between the two methods were calculated using SPSS version 26.0 software (https://www.ibm.com/cn-zh/spss, accession on 13 June 2023).

## 3. Results

### 3.1. Construction of the Standard Plasmids

The targeted fragments of the ASFV B646L, MGF505-2R and I177L genes were amplified via PCR, purified, ligated into the pMD18-T vector and transformed into DH5α competent cells. The positive clones were cultured, the recombinant plasmids were extracted, and they were confirmed by sequencing. The initial concentrations of the standard plasmids, which were named pASFV-B646L-1, pASFV-MGF505-2R-1 and pASFV-I177L-1, were determined to be 2.16 × 10^10^, 2.0 × 10^10^ and 3.45 × 10^10^ copies/µL, respectively. All standard plasmids were diluted to 2.0 × 10^10^ copies/µL and stored at −80 °C until use.

### 3.2. Determination of the Optimal Parameters

After optimization, the optimal annealing temperature, concentrations of the primers and probes, and amplification cycles were determined for the triplex qPCR assay. The reaction system in a total volume of 25 µL is shown in Table 2. The amplification parameters were as follows: 95 °C for 10 s; 40 cycles of 95 °C for 5 s; and 59 °C for 30 s. Samples with a Ct value of ≤36 were considered to be positive samples, and samples with a Ct value of >36 were considered to be negative samples.

**Figure 1:**
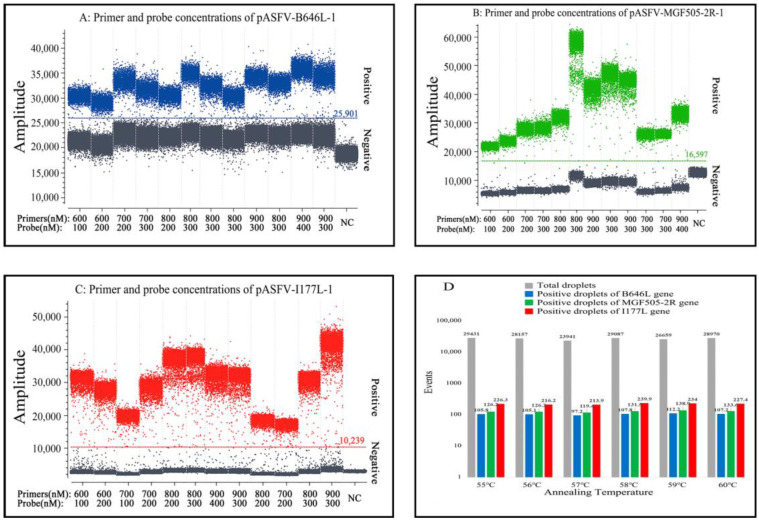
Optimization of the primer and probe concentrations (**A**–**C**) and the annealing temperature (**D**) for triplex cdPCR. (**A**–**C**) Amplification results of pASFV-B646L-1, pASFV-MGF505-2R-1 and pASFV-I177L-1 plasmids (all at final reaction concentrations of 2.0 × 10^4^ copies/µL) with different probe and primer concentrations. NC: Negative control. (**D**) Amplification results of pASFV-B646L-1, pASFV-MGF505-2R-1 and pASFV-I177L-1 plasmids (all at final reaction concentrations of 2.0 × 10^4^ copies/µL) with different annealing temperatures.

After optimization, the optimal annealing temperature, concentrations of the primers and probes, and amplification cycles were determined for the triplex cdPCR assay (Figure 1). The optimal reaction system in a total volume of 25 µL was obtained (Table 2). The amplification parameters were as follows: 95 °C for 10 s; 45 cycles of 95 °C for 5 s; and 59 °C for 30 s. After amplification, the Naica^TM^ system (Stilla Technologies^TM^, Villejuif, France) automatically reported the absolute concentration of each sample.

### 3.3. Generation of the Standard Curves

**Figure 2:**
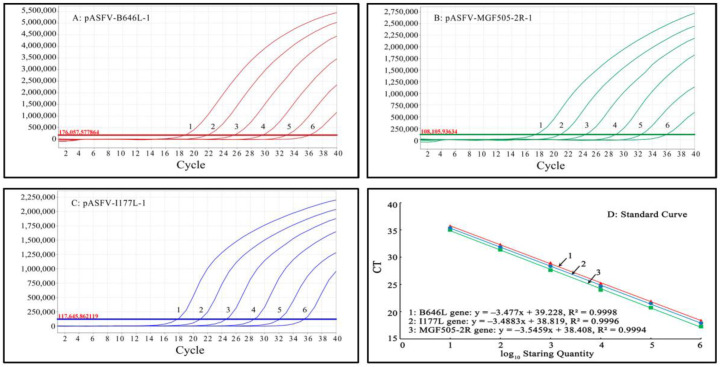
Generation of the standard curves of triplex qPCR. Amplification curves (**A**–**C**) and standard curves (**D**) of triplex qPCR. (**A**–**C**) Final reaction concentrations of curves 1 to 6 range from 2.0 × 10^6^ to 2.0 × 10^1^ copies/μL; 8: Negative control.

Mixtures of three standard plasmids, pASFV-B646L-1, pASFV-MGF505-2R-1 and pASFV-I177L-1, ranging from 2.0 × 10^7^ copies/μL to 2.0 × 10^2^ copies/μL (final reaction concentration: 2.0 × 10^6^ copies/μL to 2.0 × 10^1^ copies/μL), were used as templates to generate the standard curves of the developed qPCR. The results indicate that the slope, R^2^ and Eff% were -3.477, 0.9998 and 93.9%, respectively, for the B646L gene; -3.4883, 0.9996 and 93.5%, respectively, for the MGF505-2R gene; and -3.5459, 0.9994 and 91.4%, respectively, for the I177L gene, indicating excellent correlation coefficients (R^2^) between the initial concentrations of the template and the Ct values (Figure 2).

**Figure 3:**
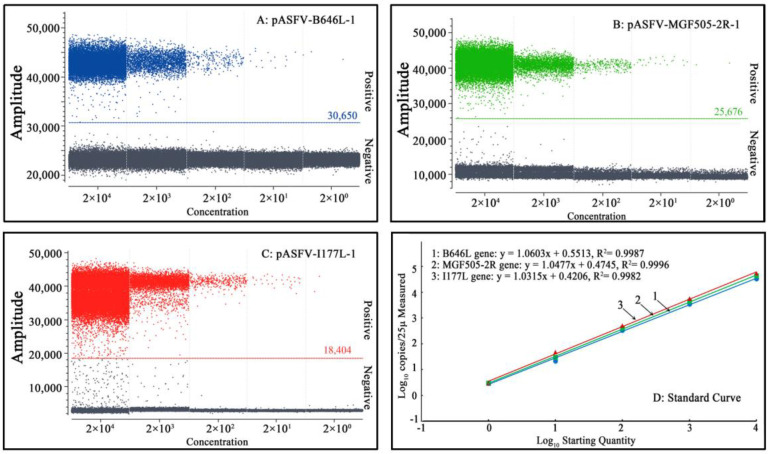
Generation of the standard curves of triplex cdPCR. (**A**–**C**) Final reaction concentrations of the plasmids range from 2.0 × 10^4^ to 2.0 × 10^0^ copies/μL. (**D**) shows the standard curves.

Mixtures of three standard plasmids, pASFV-B646L-1, pASFV-MGF505-2R-1 and pASFV-I177L-1, ranging from 2.0 × 10^5^ copies/μL to 2.0 × 10^1^ copies/μL (final reaction concentration: 2.0 × 10^4^ copies/μL to 2.0 × 10^0^ copies/μL), were used as templates to generate the standard curves of the developed triplex cdPCR. The results indicate that the slope and R^2^ were 1.0603 and 0.9987, respectively, for the B646L gene; 1.0477 and 0.9996, respectively, for the MGF505-2R gene; and 1.0315 and 0.9982, respectively, for the I177L gene, indicating excellent correlation coefficients (R^2^) between the initial concentrations of the template and the positive droplets (Figure 3).

### 3.4. Sensitivity Analysis

**Figure 4:**
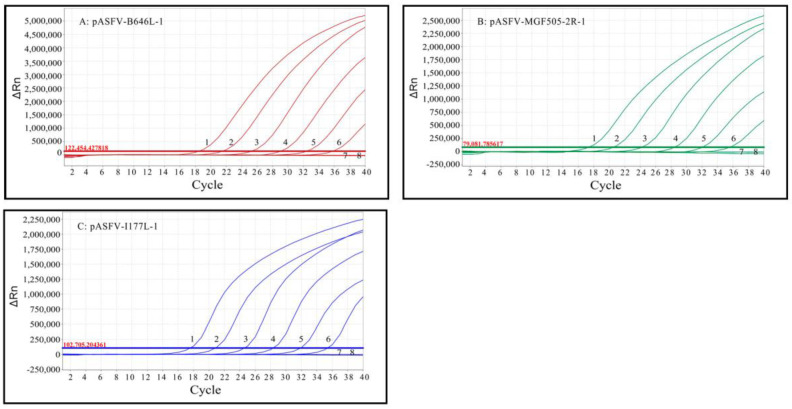
Sensitivity analysis of triplex qPCR. (**A**–**C**) Final reaction concentrations of curves 1 to 7 range from 2.0 × 10^6^ to 2.0 × 10^0^ copies/μL; 8: Negative control.

The limit of detection (LOD) of triplex qPCR was evaluated using a mixture of three standard plasmids: pASFV-B646L-1, pASFV-MGF505-2R-1 and pASFV-I177L-1, ranging from 2.0 × 10^7^ copies/μL to 2.0 × 10^1^ copies/μL (final reaction concentration: 2.0 × 10^6^ copies/μL to 2.0 × 10^0^ copies/μL). The results indicate that the LOD of pASFV-B646L-1, pASFV-MGF505-2R-1 and pASFV-I177L-1 was 20 copies/μL (Figure 4), which was equal to 500 copies/reaction.

**Figure 5:**
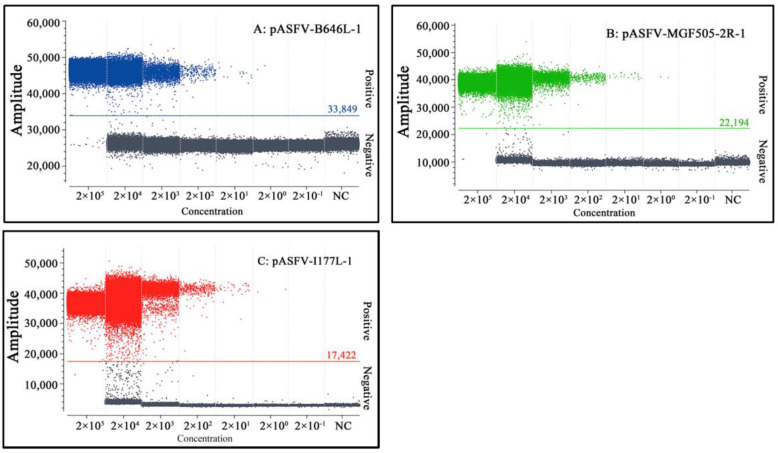
Sensitivity analysis of triplex cdPCR. (**A**–**C**) Final reaction concentrations of the plasmids range from 2.0 × 10^5^ to 2.0 × 10^−1^ copies/μL. NC: Negative control.

The LOD of triplex cdPCR was evaluated using a mixture of three standard plasmids: pASFV-B646L-1, pASFV-MGF505-2R-1 and pASFV-I177L-1, ranging from 2.0 × 10^6^ copies/μL to 2.0 × 10^0^ copies/μL (final reaction concentration: 2.0 × 10^5^ copies/μL to 2.0 × 10^−1^ copies/μL). The results revealed a gradual decline in the number of positive droplets with decreasing plasmid concentrations. The LOD of pASFV-B646L-1, pASFV-MGF505-2R-1 and pASFV-I177L-1 was 2 copies/μL (Figure 5), and the actual concentration value was revised as 12 copies/reaction via the Poisson distribution. The results demonstrated that triplex cdPCR was 42 times more sensitive than triplex qPCR.

### 3.5. Specificity Analysis

**Figure 6:**
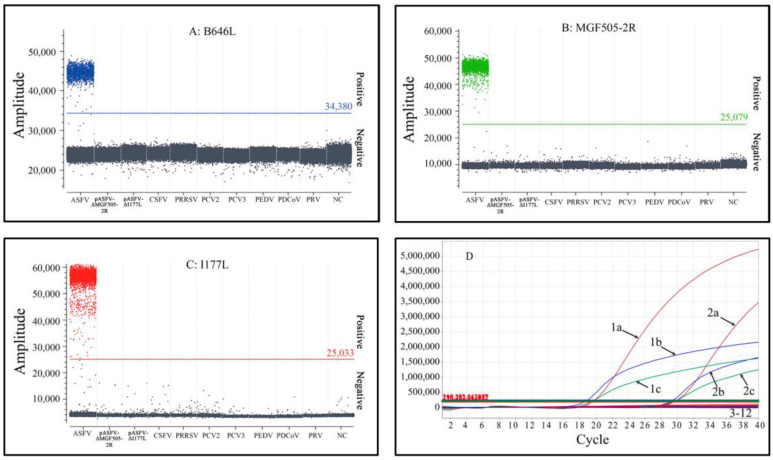
Specificity analysis of triplex cdPCR (**A**–**C**) and triplex qPCR (**D**). (**A**–**C**) Results of the specificity tests of the B646L gene (**A**), the MGF505-2R gene (**B**) and the I177L gene (**C**). NC: Negative control. (**D**) Results of the specificity test of triplex qPCR. 1a: pASFV-B646L-1; 1b: pASFV-I177L-1; 1c: pASFV-MGF505-2R-1; 2a: ASFV B646L gene; 2b: ASFV I177L gene; 2c: ASFV MGF505-2R gene; 3: pASFV-ΔI177L; 4: pASFV-ΔMGF505-2R; 5: CSFV; 6: PRRSV; 7: PCV2; 8: PCV3; 9: PEDV; 10: PDCoV; 11: PRV; 12: Negative control.

The specificity of triplex cdPCR and triplex qPCR was validated using the DNA/cDNA of ASFV, PCV2, PCV3, PRV, PRRSV, CSFV, PEDV and PDCoV, as well as the recombinant plasmids pASFV-ΔEP402R and pASFV-ΔI177L. The results indicate that the assays only generated positive droplets from ASFV, without cross-reaction with other swine viruses, demonstrating the strong specificity of the two assays (Figure 6).

### 3.6. Repeatability and Reproducibility Analysis

The repeatability and reproducibility of triplex cdPCR and triplex qPCR was evaluated using mixtures of three standard plasmids with final reaction concentrations of 2.0 × 10^4^ copies/μL, 2.0 × 10^3^ copies/μL and 2.0 × 10^2^ copies/μL. The results indicate that the intra-assay and inter-assay CVs ranged from 1.0% to 2.7% for triplex cdPCR and 0.6% to 1.8% for triplex qPCR (Table 3), indicating excellent repeatability for both developed assays.

### 3.7. Application for the Detection of Clinical Samples

A total of 1510 clinical tissue samples collected in Guangxi province, southern China, from January 2022 to December 2022 were tested via triplex cdPCR and triplex qPCR. The results show that the positivity rates of ASFV and the MGF505-2R gene-deleted ASFV strain were 14.17% (214/1510) and 0.33% (5/1510) with triplex cdPCR and 12.98% (196/1510) and 0.33% (5/1510) with triplex qPCR, respectively. No positive sample of the I177L gene-deleted ASFV strain was found with the two methods (Table 4). The coincidence rate between the two methods was 98.81% (Table 5).

The 1510 tissue samples showed a 14.17% positivity rate, of which the positivity rates of slaughterhouses, famers’ markets and harmless disposal sites were 12.12% (115/949), 16.56% (25/151) and 18.05% (74/410), respectively. The chi-squared test showed that the positivity rate of harmless disposal sites (18.05%) was significantly higher than that of slaughterhouses (12.12%) (*p* < 0.05), and the positivity rate of famers’ markets (16.56%) was between those of harmless disposal sites (18.05%) and slaughterhouses (12.12%) but showed no significant difference from them (*p* > 0.05).

## 4. Discussion

ASF has caused huge economic losses in the pig industry around the world. Since ASFV was first discovered in China in August 2018, virulent genotype II ASFV strains have rapidly spread to most provinces of China and have become the dominant epidemic strains [9,17,49]. The highly virulent genotype II ASFV strain has a nearly 100% fatality rate in infected domestic pigs and wild boars, underscoring the significant threat that the genotype II strain poses to the pig industry [50]. Naturally gene-deleted strains of ASFV, of which the MGF505 gene was the common deleted gene, have also been discovered in pig herds and have caused economic losses in the pig industry [17,21,22]. Today, the development of effective vaccines is one of the most urgent measures for the prevention and control of ASF. Gene-deleted strains of ASFV are promising vaccine candidates. Several reports have demonstrated that candidate vaccine strains, which were developed through the deletion of seven genes from the MGF360 to MGF505 families (ASFV-ΔMGF) or the I177L gene (ASFV-ΔI177L), exhibit protective effects against highly virulent ASFV strains [23,24,25,26,27,28,29]. The wild-type strains and the gene-deleted strains of ASFV have become the focus of attention. Therefore, it is necessary to monitor the wild-type and gene-deleted strains of ASFV, especially the MGF505-2R and the I177L gene-deleted strains. To date, several qPCR methods have been developed for the detection and differentiation of the wild-type strains and the gene-deleted strains of ASFV [45,46,47,48], and dPCR has also been developed for the detection of ASFV [34,35,36]. However, no multiplex cdPCR has been developed for the detection and differentiation of the wild-type strain and the MGF505-2R and/or I177L gene-deleted strains of ASFV until now.

cdPCR is a unique droplet-based technology that enables the partitioning of PCR reaction liquids into a single-layer droplet 2D array for PCR amplification, and the sample is read by a three-color fluorescence scanning device to obtain accurate quantitative data [51,52]. In comparison to qPCR, cdPCR offers several advantages, such as absolute quantification that is independent of standard curves, increased precision, low sensitivity to PCR inhibitors and the ability to detect low target concentrations [33,53]. In this study, after the optimization of the reaction system and conditions, triplex cdPCR and triplex qPCR were successfully developed for the detection of the ASFV B646L, MGF505-2R and I177L genes. Both methods demonstrated excellent specificity, high sensitivity and good repeatability. They specifically detected only the ASFV B646L, MGF505-2R and I177L genes, without cross-reaction with other swine viruses. The LOD of triplex cdPCR was 12 copies/reaction, whereas the LOD of triplex qPCR was 500 copies/reaction, indicating that the former was 42 times more sensitive than the latter. The intra-assay and inter-assay CVs ranged from 1.0% to 2.7% for triplex cdPCR and from 0.6% to 1.8% for triplex qPCR. Compared to triplex qPCR, triplex cdPCR is a more sensitive method for the accurate detection of very low concentrations of the wild-type strains and the MGF505-2R and I177L gene-deleted strains of ASFV. ddPCR targeting the ASFV K205R gene, established by Wu et al. [34], showed an LOD of 10 copies/reaction. Nanofluidic chip dPCR targeting the ASFV B646L gene, established by Jia et al. [37], showed an LOD of 30.1995 copies/reaction. Duplex ddPCR based on the ASFV B646L and EP402R genes, established by Zhu et al. [35], showed an LOD of 52 copies/reaction and 8.6 copies/reaction for B646L and EP402R, respectively. The triplex cdPCR developed in this study showed similar or superior sensitivity to the previous results. The results demonstrate that sensitive, efficient and specific triplex cdPCR and triplex qPCR were developed for the detection and differentiation of the wild-type strain and the MGF505-2R and I177L gene-deleted strains of ASFV.

A total of 1510 clinical samples collected from Guangxi province from January 2022 to December 2022 were tested with the developed triplex cdPCR. The results show that the positivity rates of the wild-type strain and the MGF505-2R gene-deleted strain of ASFV were 14.17% and 0.33%, and no positive sample of the I177L gene-deleted strain was found. These findings suggest that both the wild-type strains and the MGF505-2R gene-deleted strains of ASFV were prevalent in China, and no I177L gene-deleted ASFV strain was discovered. Compared with the results of our previous reports [17,20,36,46,54], the positivity rate of ASFV in pig herds has gradually decreased in recent years. The results indicate that important measures, such as early detection and diagnosis, the eradication of pathogenic-positive pigs and strict biosafety, which have been implemented in China in recent years [55,56], are very effective and successful measures for the prevention and control of ASF in China. However, the wild-type strains and naturally gene-deleted strains of ASFV are still circulating in some pig herds, and more and more man-made gene-deleted vaccine strains will be generated in the future. Therefore, new challenges will be confronted for the prevention and control of the disease. The developed assays provide rapid, sensitive and accurate methods to continuously monitor the circulating strains in the field in order to effectively prevent and control ASF and decrease the economic losses of ASF.

Fortunately, the I177L gene-deleted ASFV strain has not been found in the field in Guangxi province of China until now. Guangxi province is adjacent to Vietnam. Every year, there are a large number of travelers and the trade of goods between China and Vietnam, and animals and animal products are often smuggled across the border. The risk of the I177L gene-deleted ASFV strain being introduced into China from Vietnam is very high. The I177L gene-deleted vaccine can be legally used in Vietnam, but its use in China is illegal and strictly prohibited. Therefore, it is necessary to strengthen the monitoring of the I177L gene-deleted strain so that, if this strain appears in China, it can be found in a timely manner, and effective response measures can be taken. The triplex cdPCR and triplex qPCR established in this study can provide sensitive, specific and accurate methods for the detection of this strain.

## 5. Conclusions

Triplex cdPCR and triplex qPCR were successfully developed for the detection and differentiation of the wild-type strain and the MGF-505-2R and I177L gene-deleted strains of ASFV. The assays demonstrated high specificity, sensitivity and repeatability and could offer reliable methods for evaluating ASFV in clinical samples. In addition, both wild-type strains and MGF505-2R gene-deleted strains of ASFV were found in Guangxi province, southern China.

## Figures and Tables

**Table 1:** Primer and probe sequences.

Targeted Gene	Name	Sequences (5′→3′)	Amplicon (bp)
B646L	ASFV-B646L-F	GGCGTATAAAAAGTCCAGGAAATTC	79
	ASFV-B646L-R	TTCGGCGAGCGCTTTATC	
	ASFV-B646L-P	FAM-TCACCAAATCCTTTTGCGATGCAAGCT-BHQ1	
MGF505-2R	ASFV-MGF505-F	AGTCATGCACGGCATATACAA	153
	ASFV-MGF505-R	GGTTTAAACCGTGCCACATCC	
	ASFV-MGF505-P	VIC-ACGCGGCCACCCAATTCAGAGAC-BHQ1	
I177L	ASFV-I177L-F	GGCATAATTATCAAATGCGAAGGG	122
	ASFV-I177L-R	TGGAAAGTTAATGATCAGGGCTT	
	ASFV-I177L-P	Cy5-AATCCTAGCTTGCCGGTAATGGCT-BHQ2	

**Table 2:** Optimal reaction system of triplex cdPCR and triplex qPCR.

Reagent	Triplex cdPCR		Triplex qPCR	
	Volume (μL)	Final Concentration (nM)	Volume (μL)	Final Concentration (nM)
PerfeCta Multiplex qPCR ToughMix (2×)	12.5	1×	/	/
Fluorescein Sodium Salt (1 µM)	2.5	100	/	/
Premix Ex Taq (Probe qPCR) (2×)	/	/	12.5	1×
ASFV-B646L-F (25 µM)	0.8	800	0.3	300
ASFV-B646L-R (25 µM)	0.8	800	0.3	300
ASFV-B646L-P (25 µM)	0.3	300	0.3	300
ASFV-MGF505-2R-F (25 µM)	0.8	800	0.4	400
ASFV-MGF505-2R-R (25 µM)	0.8	800	0.4	400
ASFV-MGF505-2R-P (25 µM)	0.3	300	0.4	400
ASFV-I177L-F (25 µM)	0.8	800	0.4	400
ASFV-I177L-R (25 µM)	0.8	800	0.4	400
ASFV-I177L-P (25 µM)	0.3	300	0.4	400
Total nucleic acids	2.5	/	2.5	/
Nuclease-free distilled water	Up to 25	/	Up to 25	/

**Table 3:** Evaluation of repeatability and reproducibility.

Plasmid	Concentration (Copies/μL)	Intra-Assay for Repeatability						Inter-Assay for Reproducibility					
		Triplex cdPCR (Copies/Reaction)			Triplex qPCR (Ct)			Triplex cdPCR (Copies/Reaction)			Triplex qPCR (Ct)		
		$\bar{X}$	SD	CV (%)	$\bar{X}$	SD	CV (%)	$\bar{X}$	SD	CV (%)	$\bar{X}$	SD	CV (%)
pASFV-B646L-1	2.0 × 10^4^	33,983.33	500.83	1.47	25.33	0.19	0.75	34,083.33	780.36	2.29	25.46	0.27	1.06
	2.0 × 10^3^	3463.33	41.26	1.19	28.68	0.23	0.80	3455.00	56.35	1.63	28.48	0.40	1.40
	2.0 × 10^2^	331.67	6.29	1.90	32.20	0.36	1.12	335.00	9.01	2.69	32.07	0.59	1.84
pASFV-MGF505-2R-1	2.0 × 10^4^	43,225.00	650.00	1.50	24.44	0.17	0.70	43,418.33	698.75	1.61	24.38	0.23	0.94
	2.0 × 10^3^	4389.17	68.66	1.56	28.38	0.20	0.70	4370.83	91.66	2.10	28.68	0.30	1.05
	2.0 × 10^2^	390.83	5.20	1.33	31.53	0.31	0.98	395.00	10.00	2.53	32.03	0.52	1.62
pASFV-I177L-1	2.0 × 10^4^	54,550.00	912.41	1.67	23.66	0.21	0.89	54,041.67	1179.07	2.18	23.56	0.30	1.27
	2.0 × 10^3^	5607.50	90.93	1.62	28.28	0.24	0.85	5612.50	103.11	1.84	28.28	0.24	0.85
	2.0 × 10^2^	515.00	5.00	0.97	31.49	0.18	0.57	509.17	11.27	2.21	31.69	0.20	0.63

**Table 4:** Detection results of the clinical samples with triplex cdPCR and triplex qPCR.

Pathogen	Number	Triplex qPCR		Triplex cdPCR		Coincidence Rate (%)	Kappa
		Positive	Positive Rate (%)	Positive	Positive Rate (%)		
ASFV	1510	196	12.98	214	14.17	98.81	0.95
ASFV-ΔMGF505-2R	1510	5	0.33	5	0.33	100	/
ASFV-ΔI177L	1510	0	0	0	0	100	/

**Table 5:** Comparison of the results using triplex cdPCR and triplex qPCR.

Triplex qPCR	Triplex cdPCR			Coincidence Rate (%)	Kappa
	Positive	Negative	Total		
Positive	196	0	196	98.81	0.95
Negative	18	1296	1314		
Total	214	1296	1510		

## Data Availability

Not applicable.

## Supplementary Materials

The following supporting information can be downloaded at: https://www.mdpi.com/article/10.3390/pathogens12091092/s1, Table S1: The sequences of recombinant plasmids pASFV-ΔMGF505-2R and pASFV-ΔI177L.

## References

[1] Li Z., Chen W., Qiu Z., Li Y., Fan J., Wu K., Li X., Zhao M., Ding H., Fan S. (2022). African Swine Fever Virus: A Review. Life.

[2] Alonso C., Borca M., Dixon L., Revilla Y., Rodriguez F., Escribano J.M., ICTV Report Consortium (2018). ICTV Virus Taxonomy Profile Asfarviridae. J. Gen. Virol..

[3] Karger A., Pérez-Núñez D., Urquiza J., Hinojar P., Alonso C., Freitas F.B., Revilla Y., Le Potier M.-F., Montoya M. (2019). An Update on African Swine Fever Virology. Viruses.

[4] Wang G., Xie M., Wu W., Chen Z. (2021). Structures and Functional Diversities of ASFV Proteins. Viruses.

[5] Cwynar P., Stojkov J., Wlazlak K. (2019). African Swine Fever Status in Europe. Viruses.

[6] Sánchez-Vizcaíno J.M., Mur L., Martínez-López B. (2012). African Swine Fever: An Epidemiological Update. Transbound. Emerg. Dis..

[7] Danzetta M.L., Marenzoni M.L., Iannetti S., Tizzani P., Calistri P., Feliziani F. (2020). African Swine Fever: Lessons to Learn From Past Eradication Experiences. A Systematic Review. Front. Veter-Sci..

[8] Gaudreault N.N., Madden D.W., Wilson W.C., Trujillo J.D., Richt J.A. (2020). African Swine Fever Virus: An Emerging DNA Arbovirus. Front. Vet. Sci..

[9] Zhou X., Li N., Luo Y., Liu Y., Miao F., Chen T., Zhang S., Cao P., Li X., Tian K. (2018). Emergence of African Swine Fever in China, 2018. Transbound. Emerg. Dis..

[10] Tao D., Sun D., Liu Y., Wei S., Yang Z., An T., Shan F., Chen Z., Liu J. (2020). One year of African swine fever outbreak in China. Acta Trop..

[11] Ito S., Bosch J., Martínez-Avilés M., Sánchez-Vizcaíno J.M. (2022). The evolution of African swine fever in China: A global threat?. Front. Vet. Sci..

[12] Mighell E., Ward M.P. (2021). African Swine Fever spread across Asia, 2018–2019. Transbound. Emerg. Dis..

[13] Cadenas-Fernández E., Ito S., Aguilar-Vega C., Sánchez-Vizcaíno J.M., Bosch J. (2022). The Role of the Wild Boar Spreading African Swine Fever Virus in Asia: Another Underestimated Problem. Front. Vet. Sci..

[14] Blome S., Franzke K., Beer M. (2020). African swine fever—A review of current knowledge. Virus Res..

[15] Penrith M.-L., Van Heerden J., Heath L., Abworo E.O., Bastos A.D.S. (2022). Review of the Pig-Adapted African Swine Fever Viruses in and Outside Africa. Pathogens.

[16] Njau E.P., Domelevo Entfellner J.-B., Machuka E.M., Bochere E.N., Cleaveland S., Shirima G.M., Kusiluka L.J., Upton C., Bishop R.P., Pelle R. (2021). The first genotype II African swine fever virus isolated in Africa provides insight into the current Eurasian pandemic. Sci. Rep..

[17] Shi K., Liu H., Yin Y., Si H., Long F., Feng S. (2022). Molecular Characterization of African Swine Fever Virus From 2019-2020 Outbreaks in Guangxi Province, Southern China. Front. Vet. Sci..

[18] Qu H., Ge S., Zhang Y., Wu X., Wang Z. (2022). A systematic review of genotypes and serogroups of African swine fever virus. Virus Genes.

[19] Sun E., Huang L., Zhang X., Zhang J., Shen D., Zhang Z., Wang Z., Huo H., Wang W., Huangfu H. (2021). Genotype I African swine fever viruses emerged in domestic pigs in China and caused chronic infection. Emerg. Microbes Infect..

[20] Liu H., Shi K., Zhao J., Yin Y., Chen Y., Si H., Qu S., Long F., Lu W. (2022). Development of a one-step multiplex qRT-PCR assay for the detection of African swine fever virus, classical swine fever virus and atypical porcine pestivirus. BMC Vet. Res..

[21] Sun E., Zhang Z., Wang Z., He X., Zhang X., Wang L., Wang W., Huang L., Xi F., Huangfu H. (2021). Emergence and prevalence of naturally occurring lower virulent African swine fever viruses in domestic pigs in China in 2020. Sci. China Life Sci..

[22] Wang Z., Qi C., Ge S., Li J., Hu Y., Zhang X., Lv Y., Han N., Wu X., Wang Z. (2022). Genetic variation and evolution of attenuated African swine fever virus strain isolated in the field: A review. Virus Res..

[23] Chen W., Zhao D., He X., Liu R., Wang Z., Zhang X., Li F., Shan D., Chen H., Zhang J. (2020). A seven-gene-deleted African swine fever virus is safe and effective as a live attenuated vaccine in pigs. Sci. China Life Sci..

[24] O’Donnell V., Holinka L.G., Gladue D.P., Sanford B., Krug P.W., Lu X., Arzt J., Reese B., Carrillo C., Risatti G.R. (2015). African Swine Fever Virus Georgia Isolate Harboring Deletions of MGF360 and MGF505 Genes Is Attenuated in Swine and Confers Protection against Challenge with Virulent Parental Virus. J. Virol..

[25] Monteagudo P.L., Lacasta A., López E., Bosch L., Collado J., Pina-Pedrero S., Correa-Fiz F., Accensi F., Navas M.J., Vidal E. (2017). BA71ΔCD2: A New Recombinant Live Attenuated African Swine Fever Virus with Cross-Protective Capabilities. J. Virol..

[26] Lopez E., Bosch-Camós L., Ramirez-Medina E., Vuono E., Navas M.J., Muñoz M., Accensi F., Zhang J., Alonso U., Argilaguet J. (2021). Deletion Mutants of the Attenuated Recombinant ASF Virus, BA71ΔCD2, Show Decreased Vaccine Efficacy. Viruses.

[27] Borca M.V., Ramirez-Medina E., Silva E., Vuono E., Rai A., Pruitt S., Holinka L.G., Velazquez-Salinas L., Zhu J., Gladue D.P. (2020). Development of a Highly Effective African Swine Fever Virus Vaccine by Deletion of the I177L Gene Results in Sterile Immunity against the Current Epidemic Eurasia Strain. J. Virol..

[28] Borca M.V., Ramirez-Medina E., Silva E., Vuono E., Rai A., Pruitt S., Espinoza N., Velazquez-Salinas L., Gay C.G., Gladue D.P. (2021). ASFV-G-∆I177L as an Effective Oral Nasal Vaccine against the Eurasia Strain of Africa Swine Fever. Viruses.

[29] Tran X.H., Le T.T.P., Nguyen Q.H., Do T.T., Nguyen V.D., Gay C.G., Borca M.V., Gladue D.P. (2022). African swine fever virus vaccine candidate ASFV-G-DeltaI177L efficiently protects European and native pig breeds against circulating Vietnamese field strain. Transbound. Emerg. Dis..

[30] Liu Y., Xie Z., Li Y., Song Y., Di D., Liu J., Gong L., Chen Z., Wu J., Ye Z. (2023). Evaluation of an I177L gene-based five-gene-deleted African swine fever virus as a live attenuated vaccine in pigs. Emerg. Microbes Infect..

[31] Zhang H., Zhao S., Zhang H., Qin Z., Shan H., Cai X. (2023). Vaccines for African swine fever: An update. Front. Microbiol..

[32] Hawkins S.F.C., Guest P.C. (2017). Multiplex Analyses Using Real-Time Quantitative PCR. Methods Mol. Biol..

[33] Kuypers J., Jerome K.R. (2017). Applications of Digital PCR for Clinical Microbiology. J. Clin. Microbiol..

[34] Hindson C.M., Chevillet J.R., Briggs H.A., Gallichotte E.N., Ruf I.K., Hindson B.J., Vessella R.L., Tewari M. (2013). Absolute quantification by droplet digital PCR versus analog real-time PCR. Nat. Methods.

[35] Wu X., Xiao L., Lin H., Chen S., Yang M., An W., Wang Y., Yang Z., Yao X., Tang Z. (2018). Development and application of a droplet digital polymerase chain reaction (ddPCR) for detection and investigation of African swine fever virus. Can. J. Vet. Res..

[36] Zhu J., Jian W., Huang Y., Gao Q., Gao F., Chen H., Zhang G., Liao M., Qi W. (2022). Development and Application of a Duplex Droplet Digital Polymerase Chain Reaction Assay for Detection and Differentiation of EP402R-Deleted and Wild-Type African Swine Fever Virus. Front. Vet. Sci..

[37] Shi K., Chen Y., Yin Y., Long F., Feng S., Liu H., Qu S., Si H. (2022). A Multiplex Crystal Digital PCR for Detection of African Swine Fever Virus, Classical Swine Fever Virus, and Porcine Reproductive and Respiratory Syndrome Virus. Front. Vet. Sci..

[38] Jia R., Zhang G., Liu H., Chen Y., Zhou J., Liu Y., Ding P., Wang Y., Zang W., Wang A. (2020). Novel Application of Nanofluidic Chip Digital PCR for Detection of African Swine Fever Virus. Front. Vet. Sci..

[39] Wang A., Jia R., Liu Y., Zhou J., Qi Y., Chen Y., Liu D., Zhao J., Shi H., Zhang J. (2020). Development of a novel quantitative real-time PCR assay with lyophilized powder reagent to detect African swine fever virus in blood samples of domestic pigs in China. Transbound. Emerg. Dis..

[40] Wang Y., Xu L., Noll L., Stoy C., Porter E., Fu J., Feng Y., Peddireddi L., Liu X., Dodd K.A. (2020). Development of a real-time PCR assay for detection of African swine fever virus with an endogenous internal control. Transbound. Emerg. Dis..

[41] Zhan Y., Zhang L.-H., Lin Y., Cai Y.-F., Zou Y.-W., Hao Z.-Y., Luo Z.-H., Wang N.-D., Deng Z.-B., Yang Y. (2021). Development and preliminary testing of a probe-based duplex real-time PCR assay for the detection of African swine fever virus. Mol. Cell. Probes.

[42] Li X., Hu Y., Liu P., Zhu Z., Liu P., Chen C., Wu X. (2022). Development and application of a duplex real-time PCR assay for differentiation of genotypes I and II African swine fever viruses. Transbound. Emerg. Dis..

[43] Cao S., Lu H., Wu Z., Zhu S. (2022). A duplex fluorescent quantitative PCR assay to distinguish the genotype I and II strains of African swine fever virus in Chinese epidemic strains. Front. Vet. Sci..

[44] Gao Q., Feng Y., Yang Y., Luo Y., Gong T., Wang H., Gong L., Zhang G., Zheng Z. (2022). Establishment of a Dual Real-Time PCR Assay for the Identification of African Swine Fever Virus Genotypes I and II in China. Front. Vet. Sci..

[45] Yang H., Peng Z., Song W., Zhang C., Fan J., Chen H., Hua L., Pei J., Tang X., Chen H. (2022). A triplex real-time PCR method to detect African swine fever virus gene-deleted and wild type strains. Front. Vet. Sci..

[46] Zhao K., Shi K., Zhou Q., Xiong C., Mo S., Zhou H., Long F., Wei H., Hu L., Mo M. (2022). The Development of a Multiplex Real-Time Quantitative PCR Assay for the Differential Detection of the Wild-Type Strain and the MGF505-2R, EP402R and I177L Gene-Deleted Strain of the African Swine Fever Virus. Animals.

[47] Lin Y., Cao C., Shi W., Huang C., Zeng S., Sun J., Wu J., Hua Q. (2020). Development of a triplex real-time PCR assay for detection and differentiation of gene-deleted and wild-type African swine fever virus. J. Virol. Methods.

[48] Guo Z., Li K., Qiao S., Chen X.-X., Deng R., Zhang G. (2020). Development and evaluation of duplex TaqMan real-time PCR assay for detection and differentiation of wide-type and MGF505-2R gene-deleted African swine fever viruses. BMC Vet. Res..

[49] Ge S., Li J., Fan X., Liu F., Li L., Wang Q., Ren W., Bao J., Liu C., Wang H. (2018). Molecular Characterization of African Swine Fever Virus, China, 2018. Emerg. Infect. Dis..

[50] Ata E.B., Li Z.-J., Shi C.-W., Yang G.-L., Yang W.-T., Wang C.-F. (2022). African swine fever virus: A raised global upsurge and a continuous threaten to pig husbandry. Microb. Pathog..

[51] Madic J., Zocevic A., Senlis V., Fradet E., Andre B., Muller S., Dangla R., Droniou M.E. (2016). Three-color crystal digital PCR. Biomol. Detect. Quantif..

[52] Tan L.L., Loganathan N., Agarwalla S., Yang C., Yuan W., Zeng J., Wu R., Wang W., Duraiswamy S. (2023). Current commercial dPCR platforms: Technology and market review. Crit. Rev. Biotechnol..

[53] Whale A.S., Cowen S., Foy C.A., Huggett J.F. (2013). Methods for Applying Accurate Digital PCR Analysis on Low Copy DNA Samples. PLoS ONE.

[54] Chen Y., Shi K., Liu H., Yin Y., Zhao J., Long F., Lu W., Si H. (2021). Development of a multiplex qRT-PCR assay for detection of African swine fever virus, classical swine fever virus and porcine reproductive and respiratory syndrome virus. J. Vet. Sci..

[55] Liu Y., Zhang X., Qi W., Yang Y., Liu Z., An T., Wu X., Chen J. (2021). Prevention and Control Strategies of African Swine Fever and Progress on Pig Farm Repopulation in China. Viruses.

[56] Dixon L.K., Stahl K., Jori F., Vial L., Pfeiffer D.U. (2020). African Swine Fever Epidemiology and Control. Annu. Rev. Anim. Biosci..

