# Supplementary material for: Triplex Crystal Digital PCR for the Detection and Differentiation of the Wild-Type Strain and the MGF505-2R and I177L Gene-Deleted Strain of African Swine Fever Virus
Source: Pathogens. 2023 Aug 28;12(9):1092. doi: 10.3390/pathogens12091092 (PMC10534775; doi:10.3390/pathogens12091092)
Supplement: Supplementary file 1 [file pathogens-12-01092-s001.zip › pathogens-2505637-supplementary.pdf]

Supplementary

Table S1. The sequences of recombinant plasmids pASFV-ΔMGF505-2R and pASFV-ΔI177L.

Plasmid	Sequence
pASFV-ΔMGF505-2R	TATCCCTAAGAATATATCTTATAACTAGACTTATAGCAGTAAAAATCAACT<br>TTGGTTATTCTTTTTAATATAAAACGTCTAATTACTTGCAAAGGACTATAA<br>AGCCCATTTTCCTCAGCTAGAATTTTTATTTTTTAATGAAGTAGGGGGAT<br>AATTCAATAGATATCCATCATTAATATTGATTATATTTTCGAATATTATCTTC<br>TATGGTGCAAGATAATCATCTAGCGCGTGAAACATGTCCTCTTCTCTTCA<br>GGAACTTTGTCGAAAAAAGCTGCCT
pASFV-ΔI177L	GGGATTCTCTATCAGGTGTCTGTACTCTGCTATTAAAAACCTGGAAACC<br>ATGGTTATTTAATATTAATTAAATTCCCTGGTTTATTCCTCCTTAAAAGTA<br>GATGAACCTCTTTTGTTTTTTATTGGGTTCATTTTTACTAAATTTATGAAT<br>AAAAGATTATTATATTCGAATGTTTGTCCAATATGGACAACTTTGTCACC<br>AGATGTTACATTTGATTTGGTTGTTAGTGGCTGAAGCTTGGCACAATCA<br>AAAATAAGCCCATTAACACTAAGATATAG
